# Critical gene signature and immunological characterization in peripheral vascular atherosclerosis: novel insights from mendelian randomization and transcriptomics

**DOI:** 10.3389/fgene.2024.1361445

**Published:** 2024-04-10

**Authors:** Wei Xie, Shumin Chen, Hanqing Luo, Chuiyu Kong, Dongjin Wang

**Affiliations:** ^1^ Department of Cardiothoracic Surgery, Nanjing Drum Tower Hospital, Affiliated Hospital of Medical School, Nanjing University, Nanjing, China; ^2^ Institute of Cardiothoracic Vascular Disease, Nanjing University, Nanjing, China; ^3^ Department of Critical Care Medicine, Nanjing Drum Tower Hospital, Affiliated Hospital of Medical School, Nanjing University, Nanjing, China

**Keywords:** peripheral vascular atherosclerosis, mendelian randomization, expression quantitative trait loci, transcriptomics, gene signature, functional enrichment, immune cell infiltration

## Abstract

**Introduction:**

Peripheral vascular atherosclerosis (PVA) is a chronic inflammatory disease characterized by lipid accumulation in blood vessel walls, leading to vessel narrowing and inadequate blood supply. However, the molecular mechanisms underlying PVA remain poorly understood. In this study, we employed a combination of Mendelian randomization (MR) analysis and integrated transcriptomics to identify the critical gene signature associated with PVA.

**Methods:**

This study utilized three public datasets (GSE43292, GSE100927 and GSE28829) related to peripheral vascular atherosclerosis obtained from the Gene Expression Omnibus database. Instrumental variables (IVs) were identified through expression quantitative trait loci (eQTL) analysis, and two-sample MR analysis was performed using publicly available summary statistics. Disease critical genes were identified based on odds ratios and intersected with differentially expressed genes in the disease dataset. GSE28829 dataset was used to validate the screened disease critical genes. Functional enrichment analysis, GSEA analysis, and immune cell infiltration analysis were performed to further characterize the role of these genes in peripheral vascular atherosclerosis.

**Results:**

A total of 26,152 gene-related SNPs were identified as IVs, and 242 disease-associated genes were identified through MR analysis. Ten disease critical genes (ARHGAP25, HCLS1, HVCN1, RBM47, LILRB1, PLAU, IFI44L, IL1B, IFI6, and CFL2) were significantly associated with peripheral vascular atherosclerosis. Functional enrichment analysis using KEGG pathways revealed enrichment in the NF-kappa B signaling pathway and osteoclast differentiation. Gene set enrichment analysis further demonstrated functional enrichment of these genes in processes related to vascular functions and immune system activation. Additionally, immune cell infiltration analysis showed differential ratios of B cells and mast cells between the disease and control groups. The correlations analysis highlights the intricate interplay between disease critical genes and immune cells associated with PVA.

**Conclusion:**

In conclusion, this study provides new insights into the molecular mechanisms underlying PVA by identifying ten disease critical genes associated with the disease. These findings, supported by differential expression, functional enrichment, and immune system involvement, emphasize the role of these genes in vascular function and immune cell interactions in the context of PVA. These findings contribute to a better understanding of PVA pathogenesis and offer potential targets for further mechanistic exploration and therapeutic interventions.

## 1 Introduction

Peripheral vascular atherosclerosis (PVA) is a chronic inflammatory disease characterized by the accumulation of lipids in the intimal layer of blood vessel walls, leading to vessel narrowing and inadequate blood supply to peripheral tissues (Herrington et al., 2016; Morley et al., 2018). As the disease progresses, atherosclerotic plaques undergo fibrosis and calcification. Initially, there may be no symptoms, but individuals may experience leg pain, cramping, numbness, and difficulty walking as plaque erosion within the lumen of the blood vessels blocks normal blood circulation. In severe cases, PVA can result in tissue damage, ulceration, and even gangrene (Ibanez et al., 2021; Libby, 2021; Polonsky and McDermott, 2021). Atherosclerosis is marked by vascular inflammation, endothelial dysfunction, plaque formation, and reduced oxygen supply to target organs (Frostegård, 2013). Despite its clinical significance of these findings, the molecular mechanisms underlying PVA remain unclear, hindering our understanding of its pathogenesis.

Expression quantitative trait loci (eQTL) refer to genetic variants associated with statistical correlations between specific single nucleotide polymorphisms (SNPs) and gene expression levels, ultimately influencing a quantitative trait (Zhu et al., 2016). eQTL studies have illuminated the impact of genetic variation on complex traits and diseases by examining correlations between specific SNPs and gene expression levels (Ratnapriya et al., 2019). In contrast to observational epidemiological studies, genetic variation follows the principle of random allele assignment to offspring, akin to a randomized controlled experiment (Thanassoulis and O'Donnell, 2009). The fundamental principle behind Mendelian randomization (MR) analysis is utilizing genetic variation as an instrumental variable to evaluate causal relationships between risk factors and specific diseases (Thanassoulis and O'Donnell, 2009; Skrivankova et al., 2021).

In this article, we use MR analysis to examine the causal relationship between eQTL data of genes and PVA. By integrating transcriptomics microarray data, we aim to identify key genes robustly associated with PVA. Additionally, we utilize the immune infiltration analysis algorithm “CIBERSORT” to deepen our understanding of immune infiltration levels in PVA. We also explore the role of disease-critical genes in disease pathogenesis through the Gene Set Enrichment Analysis (GSEA). Furthermore, we evaluate the correlation between biomarkers and infiltrating immune cells, as well as the correlation between infiltrating immune cells themselves. These identified genes can be used as candidate molecules for the treatment of PVA, and further exploration of the underlying mechanisms may help to become potential targets for drug development.

## 2 Materials and methods

### 2.1 Research design and data sources

Three datasets related to peripheral vascular atherosclerosis were obtained from the NCBI Gene Expression Omnibus database (https://www.ncbi.nlm.nih.gov/geo/). The datasets used were GSE43292, GSE100927, and GSE28829. GSE43292 included 32 carotid non-atherosclerotic samples and 32 carotid atherosclerotic samples. GSE100927 dataset included 35 control arteries without AS lesions (from deceased organ donors) and 69 human peripheral arteries (carotid, femoral, and infra-popliteal arteries) with AS. GSE28829 included patients with pathological intimal thickening and advanced fibrous cap atherosclerotic carotid plaques. Detailed information on these datasets is shown in Table 1. The GSE43292 and GSE100927 datasets were combined to form a disease dataset, while GSE28829 was used as a validation set.

**TABLE 1:** Characteristics of datasets.

GEO accession	Platform	Contains	Country	Year	Contributor
GSE28829	GPL570	13 early carotid plaque patients and 16 advanced carotid plaque patients	Netherlands	2011	Marco Manca
GSE100927	GPL17077	35 control arteries without AS lesions and 69 human peripheral arteries with AS	France	2017	Marja Steenman
GSE43292	GPL6244	32 carotid non-atherosclerotic samples and 32 carotid atherosclerotic samples	France	2013	Catherine Cerutti
GWAS ID	Trait	Contains	Population	Year	Number of SNPs
finn-b-DM_PERIPHATHERO	Peripheral atherosclerosis	162201 control and 6631 cases	European	2021	16,380,247

Two-sample MR analysis was conducted using a Genome-Wide Association Study (GWAS) pooled dataset to examine the causal relationship between candidate genes and peripheral vascular atherosclerosis. Exposure data were obtained from the IEU Open GWAS project eQTL dataset for all genes, consisting of 19,942 genes. The outcome data were obtained from the IEU Open GWAS project with the GWAS ID “finn-b-DM_PERIPHATHERO,” which included data from 6,631 disease groups and 162,201 controls. The MR analyses used publicly available summary statistics, thereby not requiring additional ethical approval or informed consent.

### 2.2 Data merging and screening for differentially expressed genes (DEGs)

The GSE43292 and GSE100927 datasets were merged using the “sva” and “limma” packages (Chen et al., 2011; Ritchie et al., 2015) in R. Normalization was performed, and batch effects were corrected using the Combat tool. Principal component analysis (PCA) was used to validate the batch effects correction. The “limma” package was used to calculate differentially expressed genes (DEGs) between the disease and control groups, with a significance threshold set at *p* < 0.05 and |Fold Change| > 1.5. Volcano plots were generated by “ggplot2” package (Ito and Murphy, 2013), and heatmaps for top 50 DEGs from each dataset were plotted by R software “pheatmap” package.

### 2.3 Screening of instrumental variables (IVs)

The eQTL dataset for each gene in the IEU Open GWAS project was analyzed using the “TwoSampleMR” package (Hemani et al., 2018) to evaluate the significance of the association between single nucleotide polymorphisms (SNPs) and gene expression levels (*p*-value < 5e-08). A linkage disequilibrium culling step was applied to remove SNPs (r2 = 0.001), and a physical distance threshold of 10,000 base pairs was set to clump the highly associated SNPs, retaining those with the strongest associations with gene expression levels. The F-statistic was used to assess the validity of the selected instrumental variables, with SNPs having an F-statistic greater than 10 considered valid instrumental variables (Pierce et al., 2011).

### 2.4 Mendelian randomization analysis

Five two-sample MR analysis methods, including Inverse Variance Weighting (IVW), MR Egger, Weighted Median, Simple Mode, and Weighted Mode, were used for two-sample MR analysis. The IVW method served as the primary analytical method for assessing effect estimates. The potential causal relationship between candidate genes and peripheral vascular atherosclerosis was assessed using odds ratios (ORs). Heterogeneity was assessed using Cochran Q test, with significant heterogeneity defined as *p* < 0.05. The intercept of the MR-Egger regression was used to test whether estimates of causality were affected by pleiotropy, with a *p*-value <0.05 indicating pleiotropy (Bowden et al., 2015). Leave-one-out analyses were performed by removing each instrumental variable in turn to evaluate the impact of individual SNPs on the results. A significance threshold of *p* < 0.05 was used. The “TwoSampleMR” and “VariantAnnotation” packages (Obenchain et al., 2014; Hemani et al., 2018) were employed for the analyses.

### 2.5 Screening of disease critical genes and validation

Genes with OR > 1 were intersected with differentially upregulated genes, while genes with OR < 1 were intersected with differentially downregulated genes using the “VennDiagram” package. Genes that did not pass the pleiotropy test and *p*-value of less than 0.05 tested by the IVW method were excluded. The “forestplot” package was used to display ORs and confidence intervals for the “Inverse Variance Weighting” and “Weighted Median” methods. The GSE43292 dataset was used to validate the screened disease critical genes. The “circlize” package (Gu et al., 2014) was employed to create a circular genomic map illustrating the location of the genes on the chromosomes.

### 2.6 Enrichment and functional analysis of disease critical genes

GO functional annotation and KEGG enrichment analyses of the disease critical genes were performed using the “org.Hs.e.g.,.db” and “clusterProfiler” packages (Wu et al., 2021), respectively. These analyses aimed to investigate the potential biological processes, cellular components, and molecular functions associated with the key target genes. A significance threshold of *p* < 0.05 was applied.

### 2.7 GSEA enrichment analysis

To explore differences in enriched gene sets between high and low expression groups of key genes in the disease group, GSEA enrichment analyses were conducted using the “clusterProfiler” package. The “c2. cp.kegg.v2023.2. Hs.symbols” file from the MSigDB website (https://www.gsea-msigdb.org/gsea/) was used for the analysis (Liberzon et al., 2011).

### 2.8 Determination, assessment and correlation of infiltrating immune cells

The “CIBERSORT” package was utilized to evaluate the level of immune cell infiltration between the control and disease group based on the “LM22”file (Newman et al., 2015). The screening criterion for the results was set at *p*-value <0.05. Bar plots and box plots were used to observe the differential expression levels of the 28 infiltrating immune cell types. The “linkET” and “ggplot2” packages (Ito and Murphy, 2013) in R were utilized to analyze and visualize the correlations between the infiltrating immune cell types and the disease-related key target genes in the disease group.

### 2.9 Statistical analysis

All statistical analyses were conducted using R software v4.3.0. The comparisons were assessed through the unpaired *t*-test. ROC analysis was employed to determine the discriminatory value of marker genes. The threshold for statistical significance was set at *p* < 0.05.

## 3 Results

### 3.1 Identification of DEGs

**FIGURE 1:**
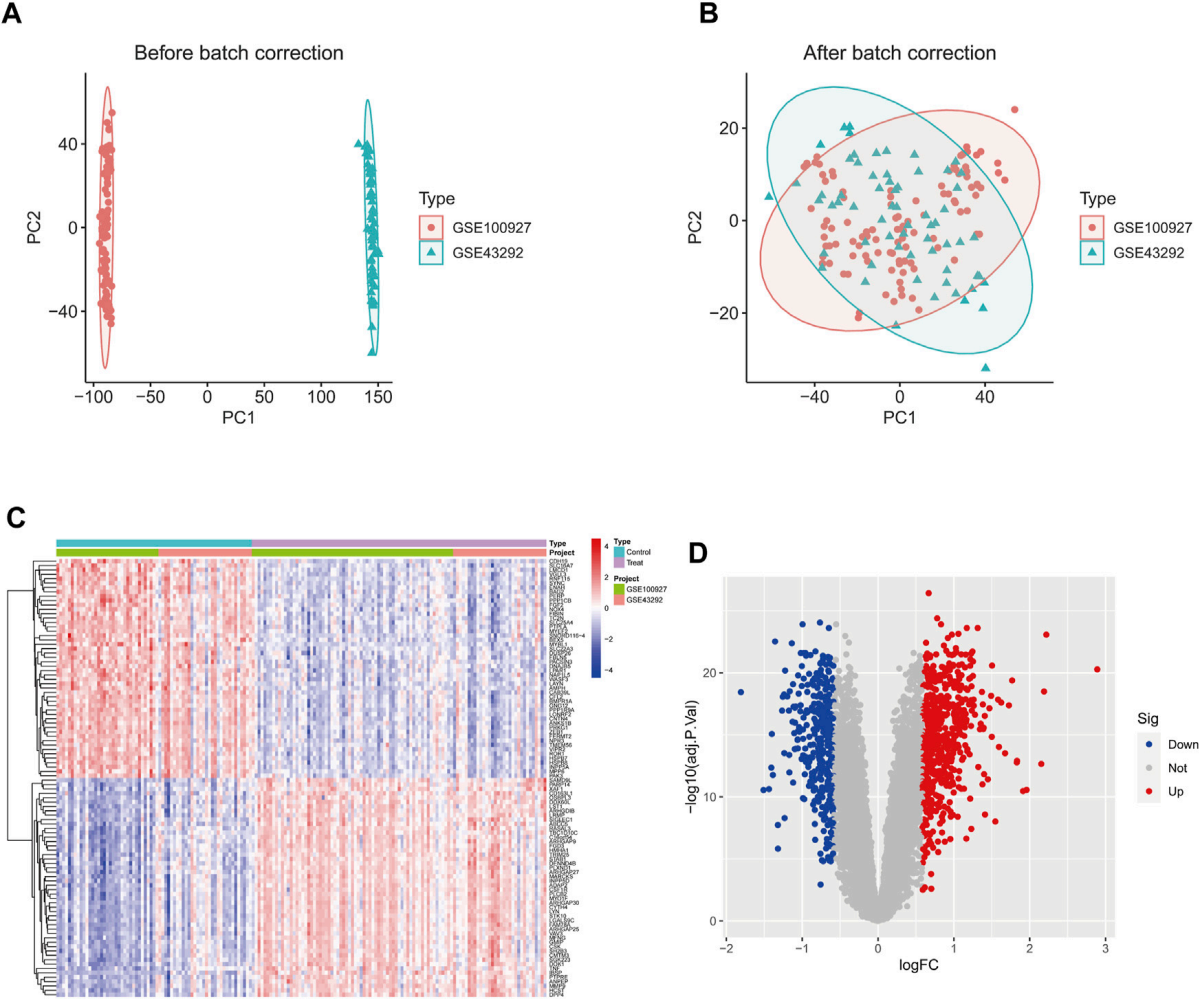
The GSE43292 and GSE100927 datasets were merged and the batch effect correction results were validated by principal components. **(A)** PCA plot before batch effect correction. **(B)** PCA plot after batch effect correction. **(D)** Heatmap of the top 50 DEGs in the dataset. **(C)** Volcano plots of DEGs in the dataset. DEGs, differentially expressed genes.

The GSE43292 and GSE100927 datasets were merged, and the batch effects correction was validated using PCA (Figures 1A, B). This analysis confirmed the successful elimination of batch-to-batch differences. Subsequently, we extracted a total of 951 differentially expressed genes from the expression matrix. Among these genes, 599 were found to be significantly upregulated, while 352 were significantly downregulated. To illustrate the differential expression patterns, heat maps (Figure 1C) and volcano maps (Figure 1D) were generated.

### 3.2 Identification of IVs and screening of disease critical genes

**FIGURE 2:**
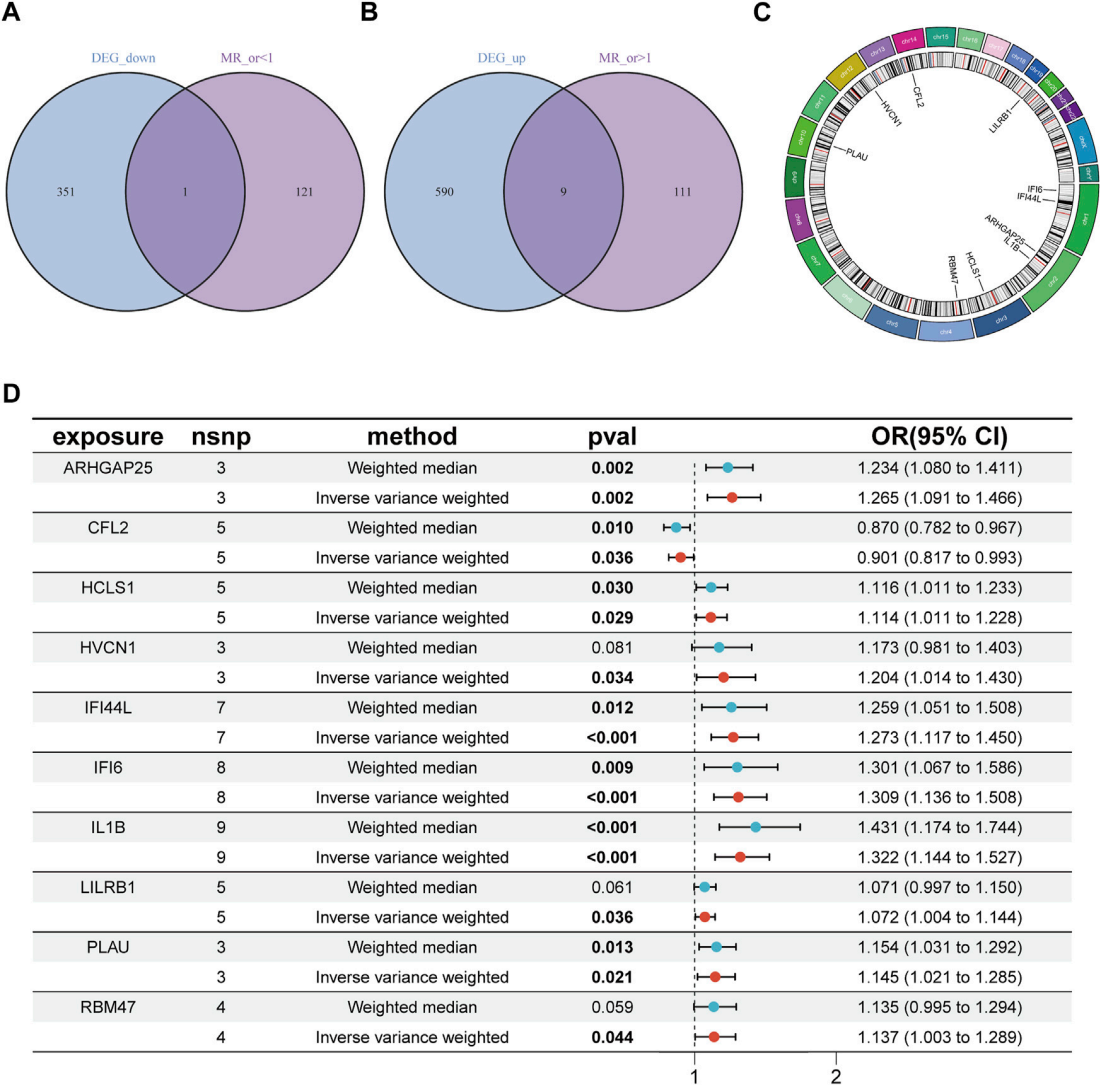
Identification of disease critical genes. **(A)** Disease downregulated DEGs are intersected with genes with OR values less than one in the MR results. **(B)** Disease upregulated DEGs are intersected with genes with OR values greater than one in the MR results. **(C)** Position of disease-critical genes on chromosomes. **(D)** Disease-critical genes are causally associated with risk of peripheral vascular atherosclerosis. DEGs, differentially expressed genes.

**FIGURE 3:**
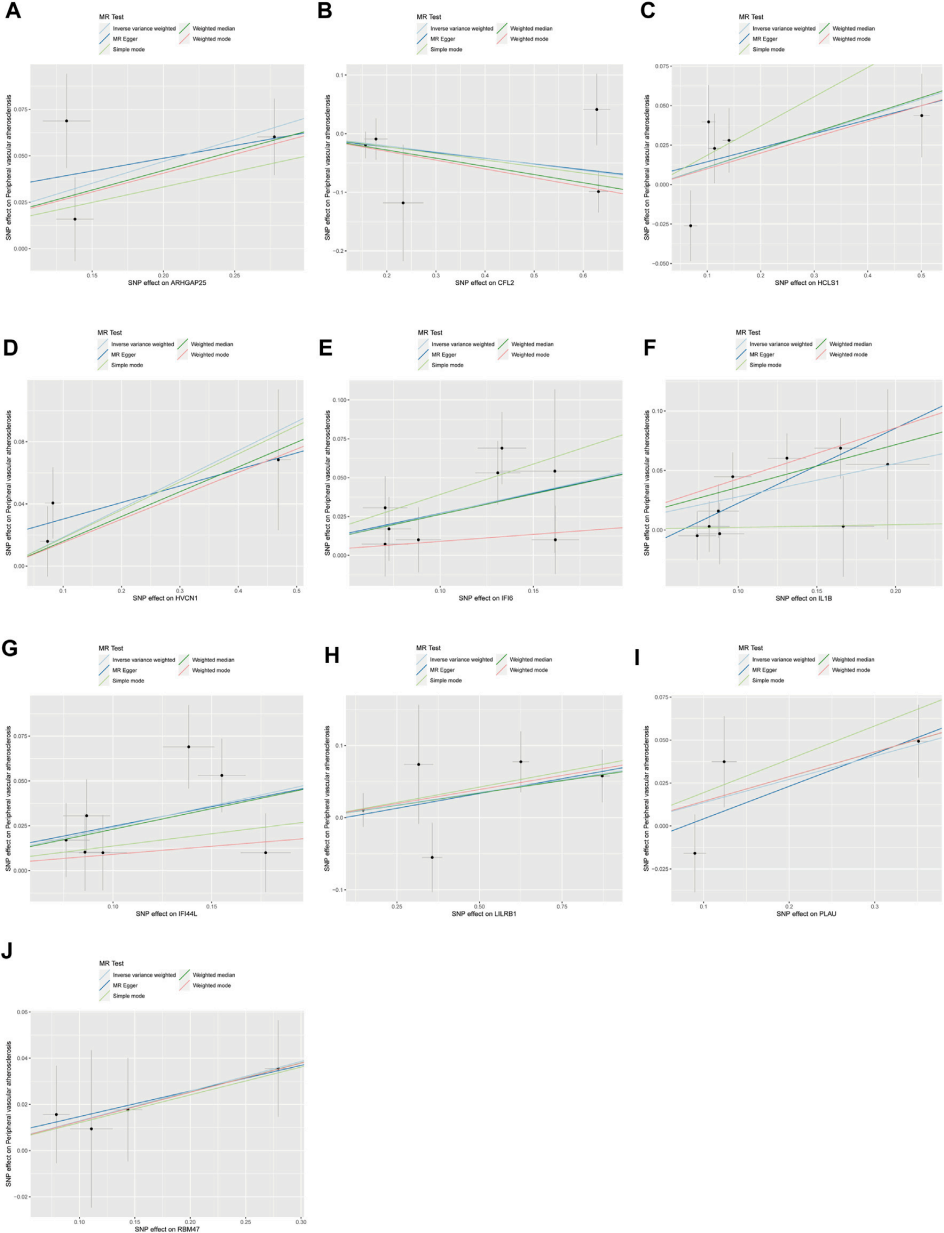
Scatterplot of MR analysis of the association between disease critical genes and peripheral vascular atherosclerosis. The slope of the line represents the estimated causal effect of the MR method. **(A–J)**: ARHGAP25, CFL2, HCLS1, HVCN1, IFI6, IL1B, IFI44L, LILRB1, PLAU and RBM47. MR, Mendelian randomization.

After the removal of consecutively unbalanced IVs, a total of 26,152 gene-related SNPs were identified as IVs based on a threshold of F > 10. Two-sample MR analysis was performed to estimate the effect of each SNP locus on peripheral vascular atherosclerosis. Sensitivity analysis was conducted to identify gene-related SNPs that met the criterion of no pleiotropy and *p* < 0.05 by IVW test, resulting in the identification of 242 disease-associated genes. The genes with odds ratios (OR) greater than one were intersected with differentially expressed upregulated genes, while the genes with OR less than one were intersected with differentially expressed downregulated genes. This screening process led to the identification of 10 disease critical genes (Figures 2A–C): ARHGAP25, HCLS1, HVCN1, RBM47, LILRB1, PLAU, IFI44L, IL1B, and IFI6, which showed significant upregulation in the disease group and were associated with an increased risk of the disease. Additionally, CFL2 was significantly downregulated and associated with a reduced disease risk (Figures 2A–D; Figures 3A–J).

### 3.3 Disease-critical gene sensitivity analyses and validation of external datasets

**FIGURE 4:**
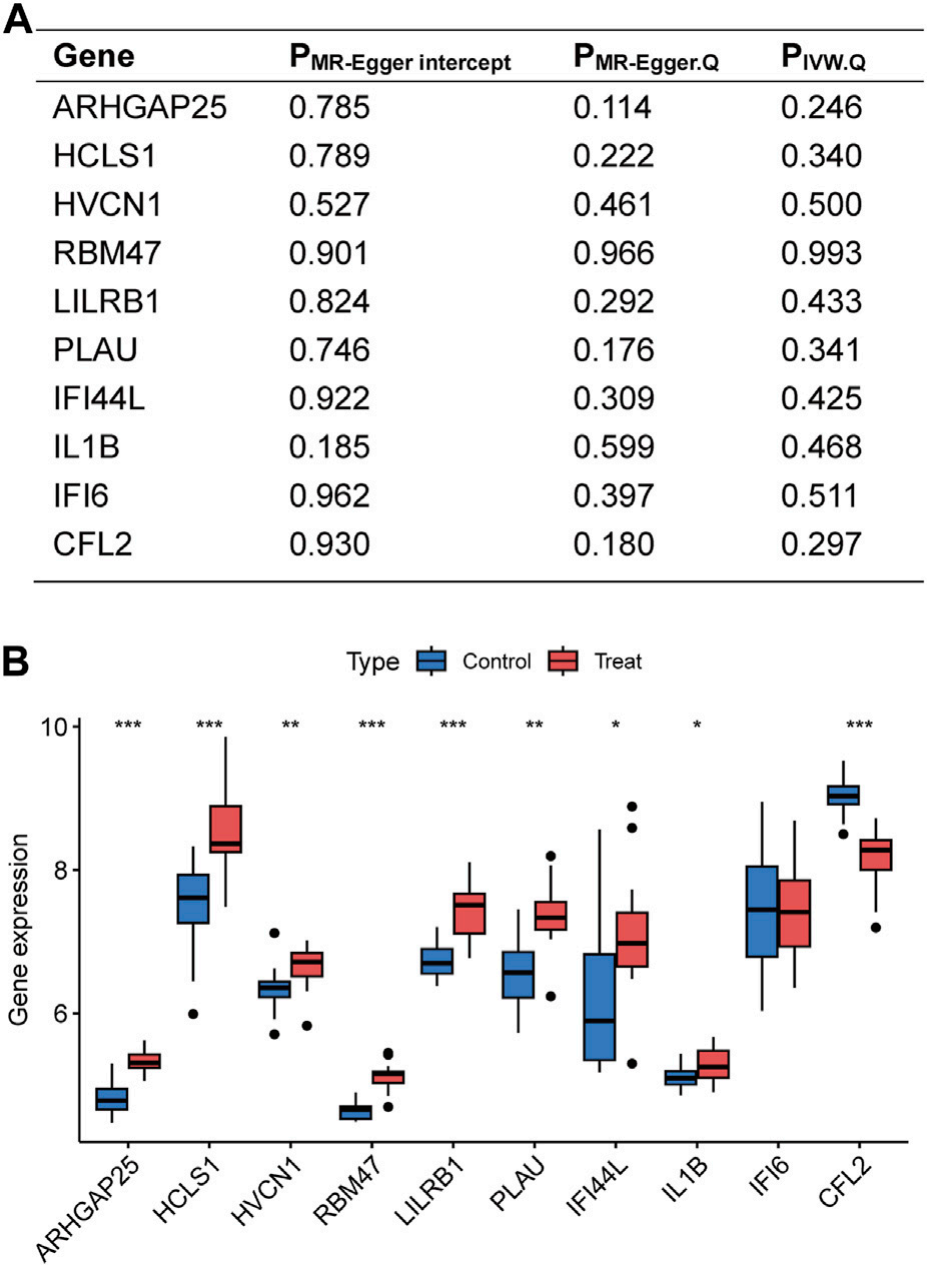
Sensitivity analyses of disease critical genes and validation of external datasets. **(A)** Intercept of MR-Egger regression was used to test whether the estimates of causality were affected by pleiotropy. Heterogeneity of results was assessed using Cochran Q test. **(B)** Expression of disease critical genes in the database GSE28829. *p < 0.05, **p < 0.01, ***p < 0.001.

**FIGURE 5:**
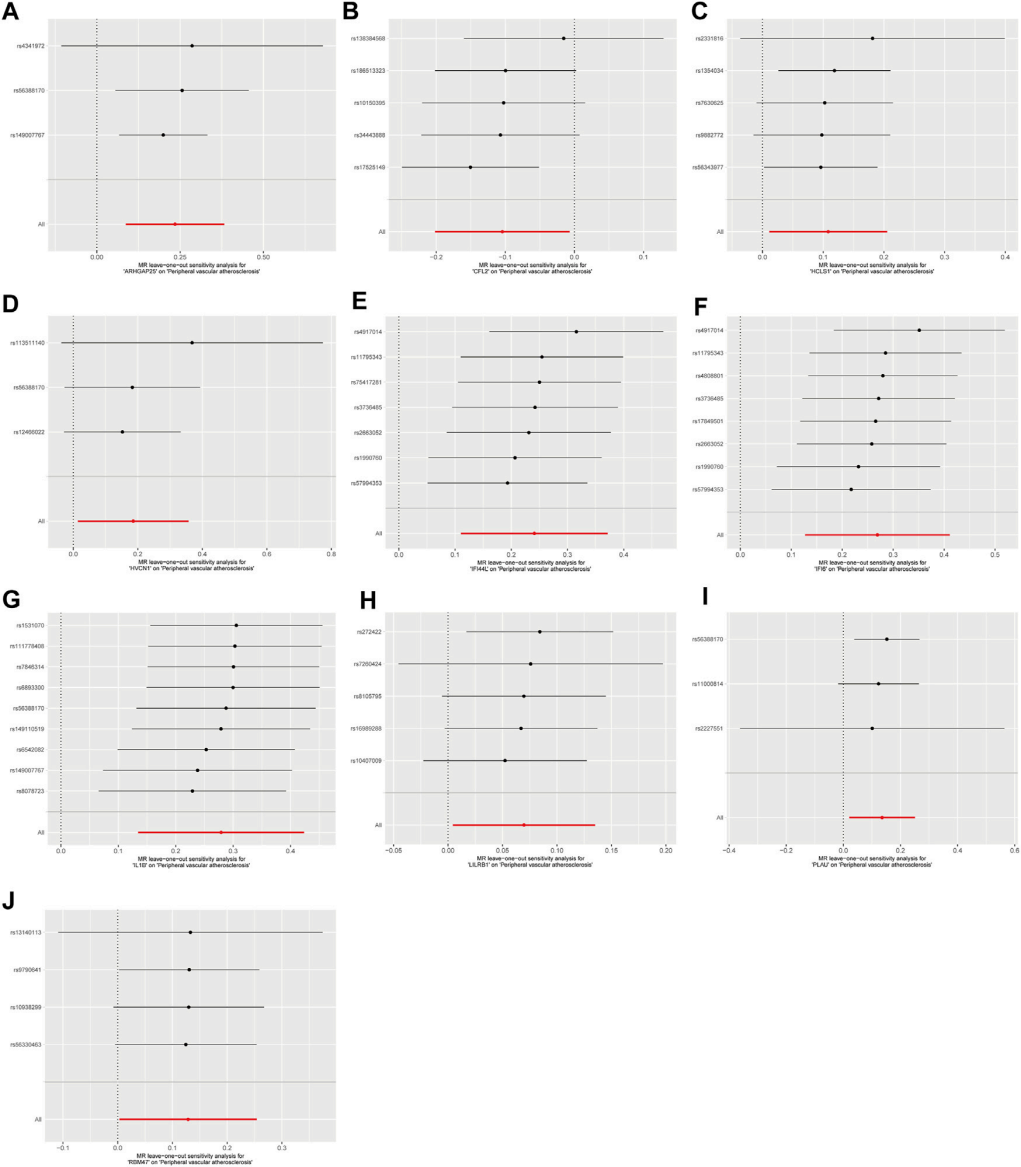
Result of “leave-one-out’ sensitivity analysis of the causal effect of disease critical genes on peripheral vascular atherosclerosis. The red line represents the estimated value of the inverse variance weighted test. **(A–J)**: ARHGAP25, CFL2, HCLS1, HVCN1, IFI6, IL1B, IFI44L, LILRB1, PLAU and RBM47.

Sensitivity analyses were performed for the 10 disease-critical genes using MR-Egger regression and the Cochran Q test. The results showed no pleiotropy and heterogeneity in any of the analyses, indicating reliable estimates (Figure 4A). To further validate the impact of each SNP locus on overall causality, leave-one-out sensitivity analyses were conducted. These analyses involved systematically removing individual SNPs and repeating the analyses to evaluate the occurrence of causality. The results showed that the estimated effects could not be attributed to any single genetic tool, as there were no significant differences in causality observed upon removal of each SNP locus (Figures 5A–J). Collectively, these findings confirm the reliability of the causal role of disease-critical genes in peripheral vascular atherosclerosis diagnosis.

The disease-critical genes were externally validated in the GSE28829 dataset. The results revealed significant expression differences in ARHGAP25, HCLS1, HVCN1, RBM47, LILRB1, PLAU, IFI44L, IL1B, and CFL2, consistent with the previous findings (Figure 4B). These validation results further strengthen the evidence that disease-critical genes exhibit significant differences in peripheral vascular atherosclerosis.

### 3.4 Functional enrichment analysis of disease critical genes

**FIGURE 6:**
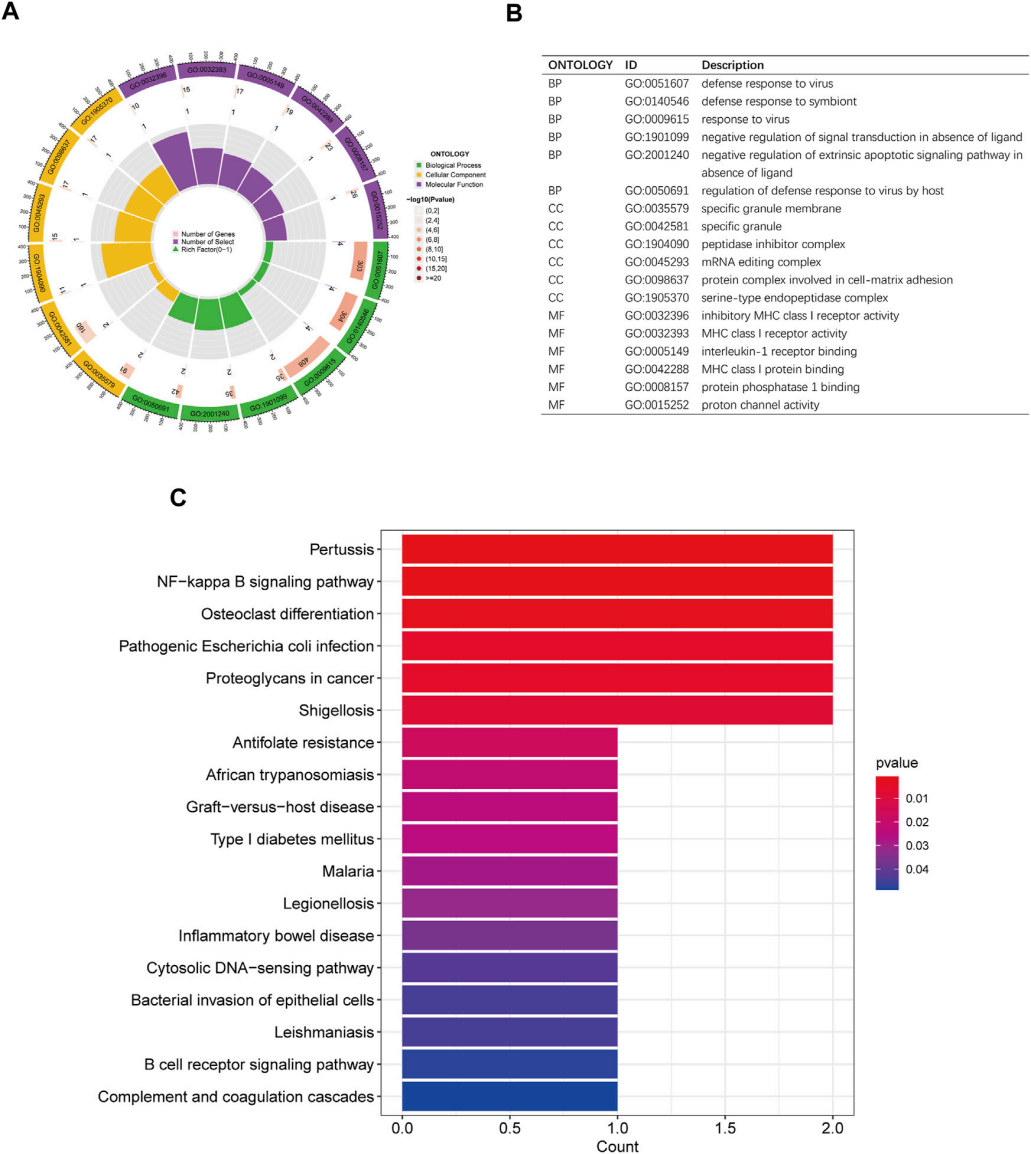
GO and KEGG enrichment analysis of disease critical genes. **(A,B)** Circle plots of GO analysis and description of biological processes, cellular components and molecular functions **(C)** KEGG analysis histogram.

Gene Ontology (GO) analysis was performed to assess the functional enrichment of disease critical genes (Figures 6A, B). The analysis encompassed three categories: biological processes, cellular components, and molecular functions. In both the disease and control groups, differentially expressed genes (DEGs) were enriched in biological processes such as defense response to virus and symbiont, as well as negative regulation of signal transduction in the absence of ligand. At the cellular component level, DEGs were enriched in specific granule membrane and peptidase inhibitor complex. Regarding molecular functions, DEGs were enriched in inhibitory MHC class I receptor activity, MHC class I receptor activity, and interleukin-1 receptor binding. Additionally, the KEGG pathway analysis revealed enrichment in the NF-kappa B signaling pathway and osteoclast differentiation (Figure 6C).

### 3.5 GSEA enrichment analysis of disease critical genes

**FIGURE 7:**
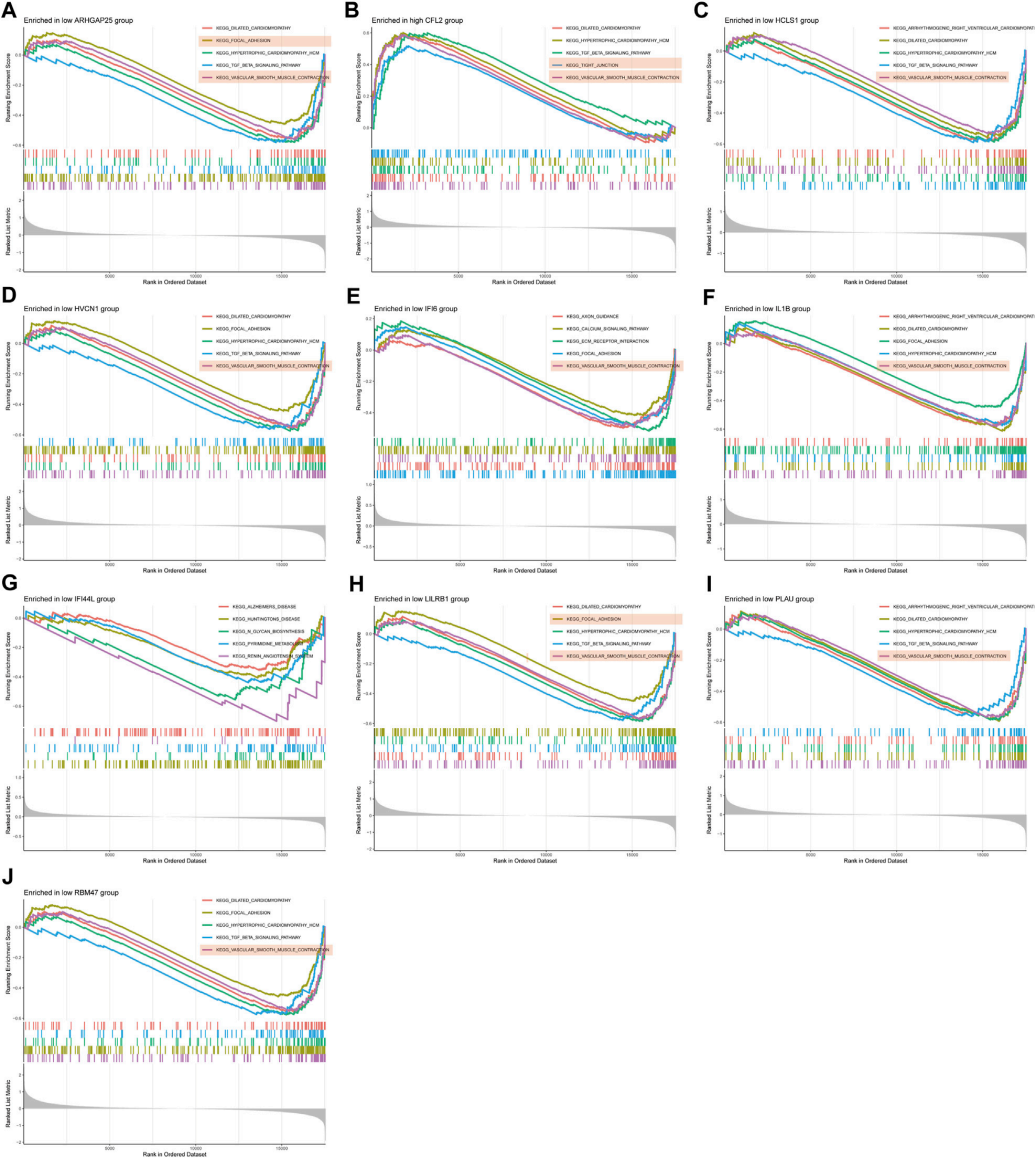
GSEA enrichment analysis of disease critical genes in peripheral vascular atherosclerotic disease. **(A,C–J)** GSEA enrichment results of low expression of ARHGAP25, HCLS1, HVCN1, IFI6, IL1B, IFI44L, LILRB1, PLAU and RBM47 **(B)** GSEA enrichment results of high expression of CFL2.

**FIGURE 8:**
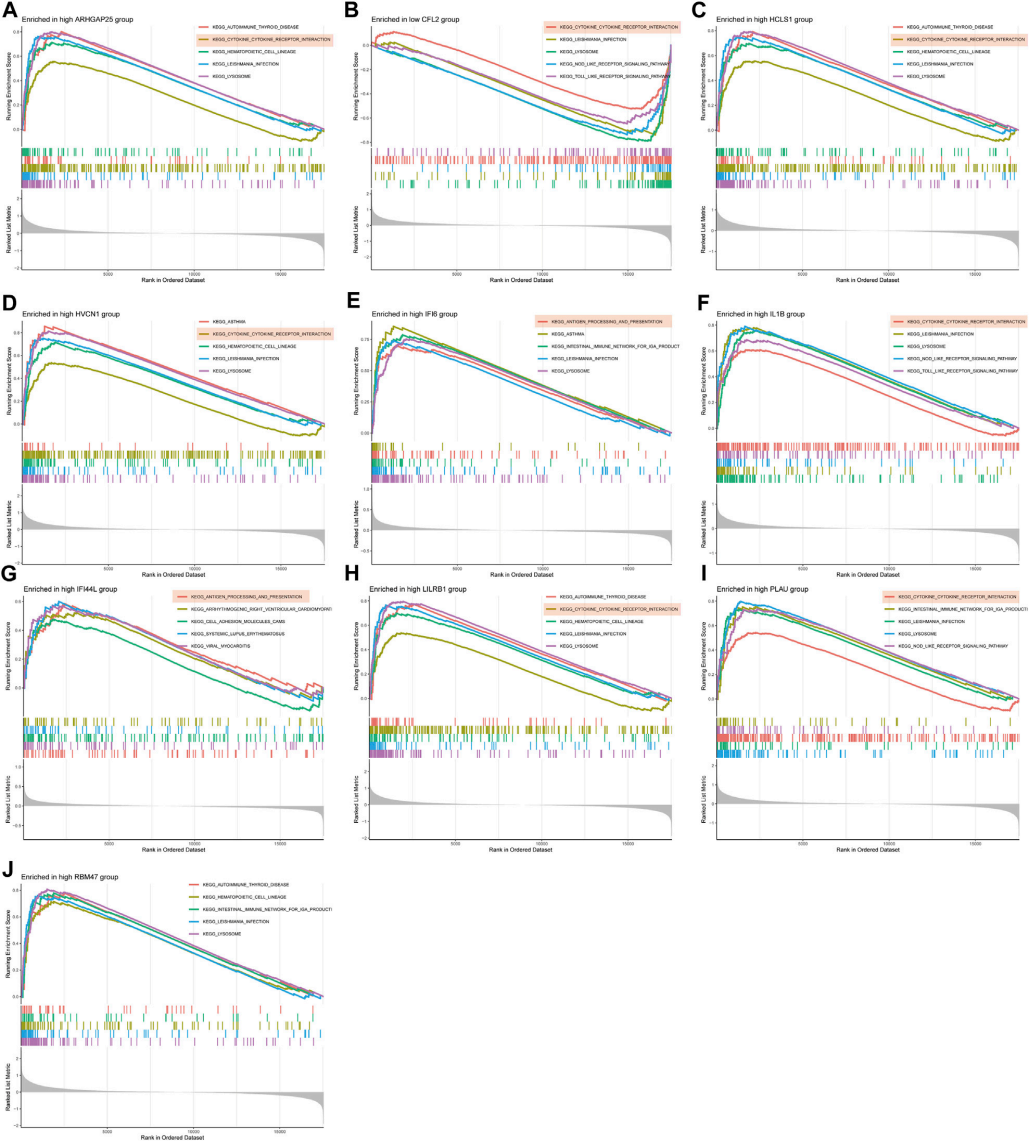
GSEA enrichment analysis of disease critical genes in peripheral vascular atherosclerotic disease. **(A,C–J)** GSEA enrichment results of high expression of ARHGAP25, HCLS1, HVCN1, IFI6, IL1B, IFI44L, LILRB1, PLAU and RBM47 **(B)** GSEA enrichment results of low expression of CFL2.

GSEA enrichment analysis was conducted to investigate the differences in enriched gene sets between the high and low expression groups of key genes in the disease group. The results demonstrated that in the disease group, the low-expression group of ARHGAP25, HCLS1, HVCN1, IFI6, IL1B, LILRB1, PLAU and RBM47 genes, as well as the high-expression group of CFL2 genes, was associated with vascular smooth muscle cell contraction, cellular adhesion, and tight junctions (Figures 7A–J). These findings suggest that alterations in these disease critical genes may contribute to the development of the disease by affecting these specific phenotypes. On the other hand, the high-expression group of ARHGAP25, HCLS1, HVCN1, IFI6, IL1B, IFI44L, LILRB1 and PLAU genes, as well as the low-expression group of CFL2 genes, were associated with cytokine-cytokine receptor interaction, antigen processing, and presentation (Figures 8A–J). These results imply that changes in disease-critical genes within the disease group may lead to changes in vascular function, immune system activation and interactions with immune cells.

### 3.6 Proportion of immune cell infiltration and correlation between disease key target genes and immune cell infiltration in disease groups

**FIGURE 9:**
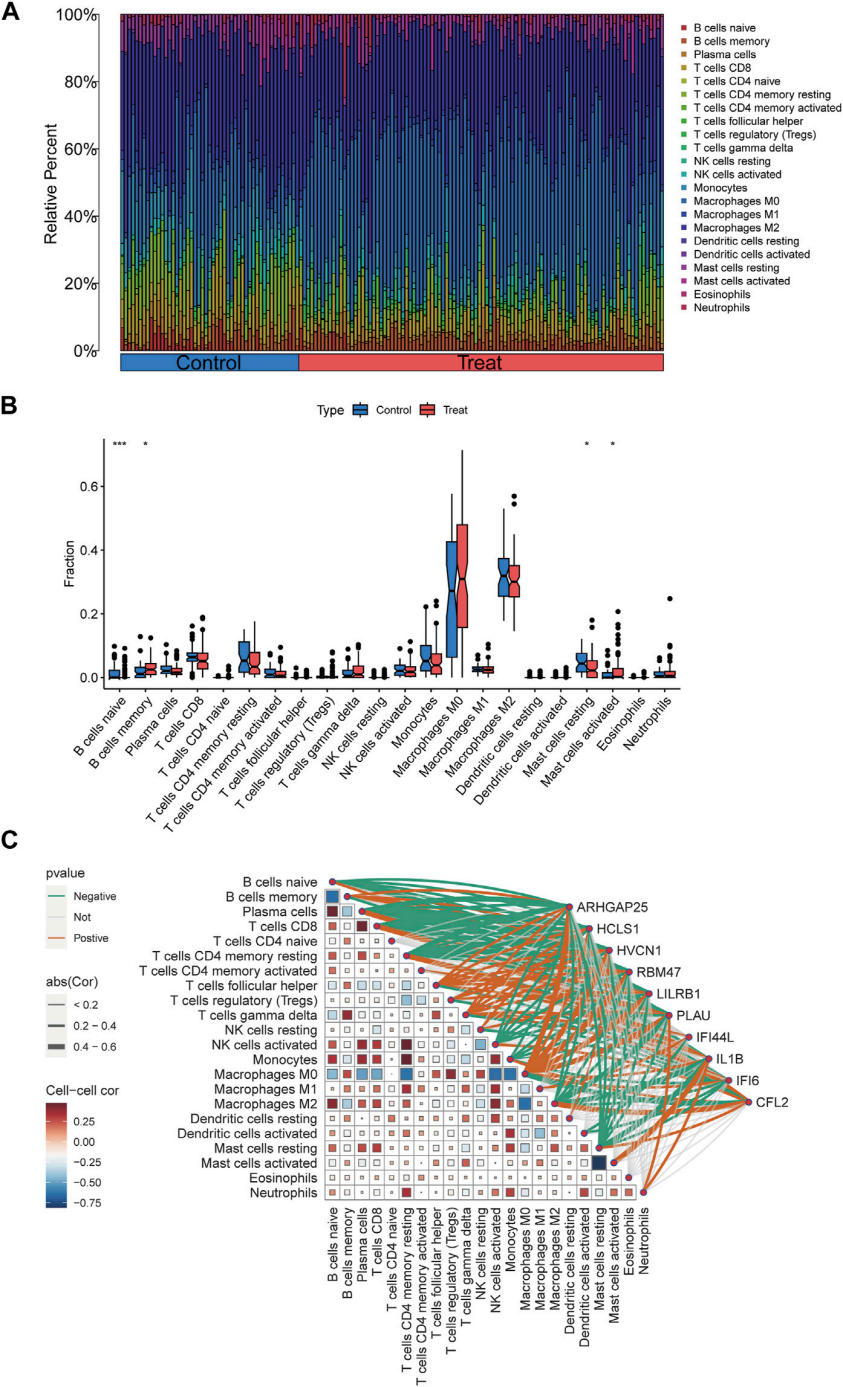
Immune infiltration analyses, correlations between disease critical genes and infiltrating immune cell types. **(A)** Bar plot displayed the relative percent of 22 infiltrated immune cells between control and PVA groups. **(B)** The differences in immune infiltration between control and PVA groups were shown in the boxplots. **(C)** Correlation analysis between disease critical genes and infiltrated immune cells. *p < 0.05, **p < 0.01, ***p < 0.001.

To further investigate the differences in immune cell infiltration between the control and disease groups, the ratio of immune cell infiltration in peripheral vascular atherosclerosis compared to non-peripheral vascular atherosclerosis was examined using the CIBERSORT algorithm. The results revealed significant changes in immune cell populations between the two groups (Figures 9A,B). Specifically, in the disease group, there was a notable decrease in the ratio of naïve B cells and resting mast cells, along with a significant increase in the ratio of memory B cells and activated mast cells. These findings suggest that B cells and mast cells may play crucial roles in the development of the disease.

Correlation analyses were conducted to explore the relationship between the 10 disease key genes and immune cells present in the disease group. Spearman correlation coefficients were calculated, demonstrating strong associations between the disease key genes and various immune cell types (Figure 9C). Notably, there was a significant negative correlation between naïve B cells and memory B cells, as well as between resting mast cells and activated mast cells. Additionally, ARHGAP25, HVCN1, LILRB1, and RBM47 were positively correlated with memory B cells, whereas CFL2 exhibited a negative correlation with memory B cells. ARHGAP25, HCLS1, HVCN1, LILRB1, PLAU, and RBM47 showed negative correlations with naïve B cells, while CFL2 displayed a positive correlation with them. Moreover, ARHGAP25, HCLS1, HVCN1, IFI6, IL1B, LILRB1, PLAU, and RBM47 exhibited negative correlations with resting mast cells, whereas CFL2 showed a positive correlation. IL1B exhibited a positive correlation with activated mast cells. These findings highlight the complex interplay between disease critical genes and immune cells associated with peripheral vascular atherosclerosis, emphasizing their significant roles in modulating the function and interactions of immune cells such as naïve B cells, memory B cells, activated mast cells, and resting mast cells.

## 4 Discussion

In this study, we utilized MR to evaluate the causal relationship between eQTL data of 19,942 genes and the risk of developing atherosclerosis in peripheral vasculature. We also employed three PVA microarray datasets (GSE43292, GSE100927, GSE28829) to identify 10 disease critical genes. To the best of our knowledge, this is the first study to investigate key disease-causing genes in peripheral vascular atherosclerosis using MR and transcriptomics. Our findings provide valuable insights into the mechanisms of peripheral vascular atherosclerosis and offer potential avenues for targeted treatment strategies.

Immune cells play a crucial role in inflammation and immune responses during the development of atherosclerosis (Roy et al., 2022). In our study, we observed significant changes in the immune infiltration landscape in patients with peripheral vascular atherosclerosis. Specifically, we found an increase in memory B cells and activated mast cells, while naive B cells and resting mast cells were significantly decreased. These findings enhance our understanding of the pathogenesis of peripheral vascular atherosclerosis. Memory B cells play a crucial role in immune memory, which is formed after the initial antigenic stimulus and enables a quick response upon encountering the same antigen again (Inoue and Kurosaki, 2023). The increase in memory B cells suggests prolonged exposure to atherosclerosis-associated antigens, leading to an intensified immune response and vascular inflammation. The decrease in naive B cells suggests a reduced ability of the immune system to respond to new antigenic stimuli, potentially affecting the appropriate response to atherosclerosis progression. Additionally, Mast cells significantly contribute to atherosclerosis by releasing pro-inflammatory cytokines and chemokines that recruit other immune cells to the site of plaque formation, thereby promoting inflammation (Shi et al., 2015). The elevation of activated mast cells and decrease in resting mast cells indicate sustained inflammatory responses and immune activation in the atherosclerotic process. These results suggest a shift towards a more activated and memory-driven immune response in PVA. However, it is essential to recognize that the composition of immune cells within the plaque of peripheral arteries can vary, and our analysis focused on specific datasets that may not fully capture the diversity of immune cell involvement in PVA. The limitations of the datasets may have restricted the detection of significant changes in macrophages and T cells, despite their known importance in atherosclerosis progression (Roy et al., 2022).

ARHGAP25 is a gene encoding for Rho GTPase activating protein 25, which plays a crucial role in cytoskeletal remodeling and leukocyte recruitment during inflammation (Csépányi-Kömi et al., 2012). HCLS1, known as hematopoietic cell-specific Lyn substrate 1, is involved in cellular signal transduction and immunomodulation. It regulates the activation and proliferation of T and B cells, thus impacting immune cell function and inflammatory responses (Taniuchi et al., 1995). HVCN1 encodes a hydrogen ion voltage-gated channel primarily expressed in immune cells. Its role in regulating immune cell function and inflammatory responses is linked to the regulation of oxidative metabolism (Capasso et al., 2010). RBM47, an RNA-binding motif protein, is associated with splicing regulation and has implications in cell migration, oxidative stress, and inflammation (Soleymanjahi et al., 2023). LILRB1 encodes an immunoglobulin-like receptor predominantly present on immune cells. Its functions encompass immune cell differentiation, proliferation, and cytokine release. It is a potential target for immune checkpoint therapy (Zeller et al., 2023). PLAU codes for plasminogen activator, urokinase, which is a secreted serine protease involved in fibrinolysis. Its activity is pivotal in cleaving fibrin into soluble peptides and facilitating fibrin removal (Urano et al., 2018). Additionally, it has also been associated with invasive phenotypes and immunosuppression (Hosen et al., 2022). IFI44L, IFI6, and IL1B are genes involved in the regulation of immune response and inflammation. They participate in immune cell activation, inflammatory regulation, and cytokine release (Dinarello, 2011; Zhang et al., 2020; Pan et al., 2023). The upregulation of these genes suggests that dysregulation in immune response, inflammation, cell migration, and gene expression may contribute significantly to atherosclerosis development and progression. In contrast, CFL2, encoding the cofilin two protein, is associated with actin depolymerization (Kremneva et al., 2014). Its role in processes such as myofiber remodeling and cell motility make it crucial in maintaining the structure and function of vascular smooth muscle cells. A decrease in CFL2 expression may impact the morphology and function of vascular smooth muscle cells, thereby influencing the development of atherosclerosis.

The positive correlations of ARHGAP25, HVCN1, LILRB1, and RBM47 with memory B cells indicate potential roles of these genes in regulating memory B cell function. LILRB1, for example, has been previously implicated in B cell regulation, where the antibody-mediated crosslinking of LILRB1 has been shown to disrupt B cell receptor activation, leading to diminished Ca2+ mobilization (Colonna et al., 1997; Zeller et al., 2023). On the other hand, CFL2’s negative correlation with memory B cells suggests a potential inhibitory role in memory B cell development or function. The negative correlations of ARHGAP25, HCLS1, HVCN1, LILRB1, PLAU, and RBM47 with naïve B cells suggest that these genes may be involved in suppressing the generation or maintenance of naïve B cells in peripheral vascular atherosclerosis. It is worth noting that HVCN1, has been shown to plays a pivotal role in regulating reactive oxygen species generation and B cell activation through its association with the B cell antigen receptor complex, impacting signaling pathways and metabolic processes crucial for B cell function (Capasso et al., 2010). The negative correlations of ARHGAP25, HCLS1, HVCN1, IFI6, IL1B, LILRB1, PLAU, and RBM47 with resting mast cells suggest potential roles of these genes in mast cell activation or migration. IL1B, a pro-inflammatory cytokine, exhibited a positive correlation with activated mast cells, which is consistent with its known role in promoting mast cell activation (Mayavannan et al., 2023). Overall, these findings highlight the intricate interplay between immune cells and gene expression in the context of peripheral vascular atherosclerosis. Further investigation into the specific roles of these genes in B cell and mast cell function in atherosclerosis is warranted to deepen our understanding of the underlying mechanisms driving the disease process and to identify potential therapeutic targets for intervention.

KEGG enrichment analysis of disease key genes revealed their involvement in the NF-kappa B signalling pathway and bone resorption differentiation. These findings unveil the crucial inflammatory pathways and cellular differentiation processes that contribute to peripheral vascular atherosclerosis. The NF-kappa B signalling pathway plays a significant role in regulating inflammation and immune responses, with its over-activation associated with chronic inflammation and vascular lesions (Mussbacher et al., 2019). Additionally, bone resorption differentiation, which corresponds to calcium deposition in atherosclerotic lesions, is related to bone metabolism (Perisic et al., 2016). Further GSEA enrichment analysis demonstrated functional differences between the low-expression and high-expression groups of disease key genes. For instance, genes such as ARHGAP25, HCLS1, HVCN1, IFI6, IL1B, LILRB1, PLAU, RBM47, when expressed at low levels and CFL2 expressed at high level, may affect processes like vascular smooth muscle cell contraction, cell adhesion, and tight junctions. These functions are crucial for regulating vasodilation, vasoconstriction, maintaining vascular wall integrity, and endothelial barrier function (Brozovich et al., 2016; Fiedler and Thum, 2018; Park-Windhol and D'Amore, 2016). Dysregulation of these functions can contribute to vascular wall damage and promote atherosclerosis progression in the peripheral vasculature (Johnson, 2014; Mundi et al., 2018). Additionally, Changes in gene expression levels of key disease genes are associated with processes such as cytokine-cytokine receptor interaction and antigen processing and presentation. These alterations may be involved in immune-related processes such as immune response, inflammatory signaling, and intracellular signal transduction, which, in turn, contribute to inflammatory reactions in the vessel wall and immune cell infiltration, facilitating the development of peripheral vascular atherosclerosis (Björkegren and Lusis, 2022). These results support the association between disease-critical genes and peripheral vascular atherosclerosis, suggesting potential mechanisms leading to vascular inflammation and the development of atherosclerosis. In-depth investigation of the functions and regulatory pathways of these key genes will aid in deciphering the important mechanisms underlying vascular inflammation and atherosclerosis, ultimately providing new therapeutic targets.

However, it is worth noting that there are a few limitations to this study. Firstly, the accuracy and reliability of instrumental variables (IVs) are highly dependent on the sample size of the GWAS, thus requiring larger sets of gene eQTL data for improved accuracy. Secondly, although MR is a powerful method, its results should be validated through further studies incorporating experimental data. Lastly, as the GWAS data primarily originated from European populations, the generalizability of our findings to other ethnicities may be limited.

## 5 Conclusion

In conclusion, our study represents the first attempt to elucidate the underlying disease mechanisms of peripheral vascular atherosclerosis using eQTL data combined with MR analysis and transcriptomics mining. Through MR analysis and microarray data mining, we have identified ten disease critical genes strongly associated with peripheral vascular atherosclerosis. These genes have the potential to serve as valuable diagnostic markers for screening and prevention in clinical practice, as well as potential targets for future mechanistic research and drug development. However, further studies are needed to validate these findings.

## Data Availability

The original contributions presented in the study are included in the article/Supplementary material, further inquiries can be directed to the corresponding authors.

## Abbreviations

ARHGAP25, Rho GTPase Activating Protein 25; HCLS1, Hematopoietic Cell-Specific Lyn Substrate 1; HVCN1, Hydrogen Voltage-Gated Channel 1; RBM47, RNA Binding Motif Protein 47; LILRB1, Leukocyte Immunoglobulin-Like Receptor B1; PLAU, Plasminogen Activator, Urokinase; IFI44L, Interferon-Induced Protein 44-Like; IL1B, Interleukin-1 Beta; IFI6, Interferon Alpha-Inducible Protein 6; CFL2, Cofilin 2.

## References

[B1] Björkegren J. L. M. Lusis A. J. (2022). Atherosclerosis: recent developments. Cell 185 (10), 1630–1645. 10.1016/j.cell.2022.04.004 35504280 PMC9119695

[B2] Bowden J. Davey Smith G. Burgess S. (2015). Mendelian randomization with invalid instruments: effect estimation and bias detection through Egger regression. Int. J. Epidemiol. 44 (2), 512–525. 10.1093/ije/dyv080 26050253 PMC4469799

[B3] Brozovich F. V. Nicholson C. J. Degen C. V. Gao Y. Z. Aggarwal M. Morgan K. G. (2016). Mechanisms of vascular smooth muscle contraction and the basis for pharmacologic treatment of smooth muscle disorders. Pharmacol. Rev. 68 (2), 476–532. 10.1124/pr.115.010652 27037223 PMC4819215

[B4] Capasso M. Bhamrah M. K. Henley T. Boyd R. S. Langlais C. Cain K. (2010). HVCN1 modulates BCR signal strength via regulation of BCR-dependent generation of reactive oxygen species. Nat. Immunol. 11 (3), 265–272. 10.1038/ni.1843 20139987 PMC3030552

[B5] Chen C. Grennan K. Badner J. Zhang D. Gershon E. Jin L. (2011). Removing batch effects in analysis of expression microarray data: an evaluation of six batch adjustment methods. PLoS One 6 (2), e17238. 10.1371/journal.pone.0017238 21386892 PMC3046121

[B6] Colonna M. Navarro F. Bellón T. Llano M. García P. Samaridis J. (1997). A common inhibitory receptor for major histocompatibility complex class I molecules on human lymphoid and myelomonocytic cells. J. Exp. Med. 186 (11), 1809–1818. 10.1084/jem.186.11.1809 9382880 PMC2199153

[B7] Csépányi-Kömi R. Sirokmány G. Geiszt M. Ligeti E. (2012). ARHGAP25, a novel Rac GTPase-activating protein, regulates phagocytosis in human neutrophilic granulocytes. Blood 119 (2), 573–582. 10.1182/blood-2010-12-324053 22096251

[B8] Dinarello C. A. (2011). A clinical perspective of IL-1β as the gatekeeper of inflammation. Eur. J. Immunol. 41 (5), 1203–1217. 10.1002/eji.201141550 21523780

[B9] Fiedler J. Thum T. (2018). Vascular smooth muscle cell remodeling. Circ. Res. 123 (12), 1261–1263. 10.1161/CIRCRESAHA.118.314184 30566049

[B10] Frostegård J. (2013). Immunity, atherosclerosis and cardiovascular disease. BMC Med. 11, 117. 10.1186/1741-7015-11-117 23635324 PMC3658954

[B11] Gu Z. Gu L. Eils R. Schlesner M. Brors B. (2014). Circlize Implements and enhances circular visualization in R. Bioinformatics 30 (19), 2811–2812. 10.1093/bioinformatics/btu393 24930139

[B12] Hemani G. Zheng J. Elsworth B. Wade K. H. Haberland V. Baird D. (2018). The MR-Base platform supports systematic causal inference across the human phenome. Elife 7, e34408. 10.7554/eLife.34408 29846171 PMC5976434

[B13] Herrington W. Lacey B. Sherliker P. Armitage J. Lewington S. (2016). Epidemiology of atherosclerosis and the potential to reduce the global burden of atherothrombotic disease. Circ. Res. 118 (4), 535–546. 10.1161/CIRCRESAHA.115.307611 26892956

[B14] Hosen S. M. Z. Uddin M. N. Xu Z. Buckley B. J. Perera C. Pang T. C. Y. (2022). Metastatic phenotype and immunosuppressive tumour microenvironment in pancreatic ductal adenocarcinoma: key role of the urokinase plasminogen activator (PLAU). Front. Immunol. 13, 1060957. 10.3389/fimmu.2022.1060957 36591282 PMC9794594

[B15] Ibanez B. Fernández-Ortiz A. Fernández-Friera L. García-Lunar I. Andrés V. Fuster V. (2021). Progression of early subclinical atherosclerosis (PESA) study: JACC focus seminar 7/8. J. Am. Coll. Cardiol. 78 (2), 156–179. 10.1016/j.jacc.2021.05.011 34238438

[B16] Inoue T. Kurosaki T. (2023). Memory B cells. Nat. Rev. Immunol. 24, 5–17. 10.1038/s41577-023-00897-3 37400644

[B17] Ito K. Murphy D. (2013). Application of ggplot2 to pharmacometric graphics. CPT Pharmacometrics Syst. Pharmacol. 2 (10), e79. 10.1038/psp.2013.56 24132163 PMC3817376

[B18] Johnson J. L. (2014). Emerging regulators of vascular smooth muscle cell function in the development and progression of atherosclerosis. Cardiovasc Res. 103 (4), 452–460. 10.1093/cvr/cvu171 25053639

[B19] Kremneva E. Makkonen M. H. Skwarek-Maruszewska A. Gateva G. Michelot A. Dominguez R. (2014). Cofilin-2 controls actin filament length in muscle sarcomeres. Dev. Cell 31 (2), 215–226. 10.1016/j.devcel.2014.09.002 25373779 PMC4223631

[B20] Libby P. (2021). The changing landscape of atherosclerosis. Nature 592 (7855), 524–533. 10.1038/s41586-021-03392-8 33883728

[B21] Liberzon A. Subramanian A. Pinchback R. Thorvaldsdóttir H. Tamayo P. Mesirov J. P. (2011). Molecular signatures database (MSigDB) 3.0. Bioinformatics 27 (12), 1739–1740. 10.1093/bioinformatics/btr260 21546393 PMC3106198

[B22] Mayavannan A. Shantz E. Haidl I. D. Wang J. Marshall J. S. (2023). Mast cells selectively produce inflammatory mediators and impact the early response to Chlamydia reproductive tract infection. Front. Immunol. 14, 1166068. 10.3389/fimmu.2023.1166068 37138882 PMC10150091

[B23] Morley R. L. Sharma A. Horsch A. D. Hinchliffe R. J. (2018). Peripheral artery disease. BMJ 360, j5842. 10.1136/bmj.j5842 29419394

[B24] Mundi S. Massaro M. Scoditti E. Carluccio M. A. van Hinsbergh V. W. M. Iruela-Arispe M. L. (2018). Endothelial permeability, LDL deposition, and cardiovascular risk factors-a review. Cardiovasc Res. 114 (1), 35–52. 10.1093/cvr/cvx226 29228169 PMC7729208

[B25] Mussbacher M. Salzmann M. Brostjan C. Hoesel B. Schoergenhofer C. Datler H. (2019). Cell type-specific roles of NF-κB linking inflammation and thrombosis. Front. Immunol. 10, 85. 10.3389/fimmu.2019.00085 30778349 PMC6369217

[B26] Newman A. M. Liu C. L. Green M. R. Gentles A. J. Feng W. Xu Y. (2015). Robust enumeration of cell subsets from tissue expression profiles. Nat. Methods 12 (5), 453–457. 10.1038/nmeth.3337 25822800 PMC4739640

[B27] Obenchain V. Lawrence M. Carey V. Gogarten S. Shannon P. Morgan M. (2014). VariantAnnotation: a Bioconductor package for exploration and annotation of genetic variants. Bioinformatics 30 (14), 2076–2078. 10.1093/bioinformatics/btu168 24681907 PMC4080743

[B28] Pan C. Hu T. Liu P. Ma D. Cao S. Shang Q. (2023). BM-MSCs display altered gene expression profiles in B-cell acute lymphoblastic leukemia niches and exert pro-proliferative effects via overexpression of IFI6. J. Transl. Med. 21 (1), 593. 10.1186/s12967-023-04464-1 37670388 PMC10478283

[B29] Park-Windhol C. D'Amore P. A. (2016). Disorders of vascular permeability. Annu. Rev. Pathol. 11, 251–281. 10.1146/annurev-pathol-012615-044506 26907525 PMC8462517

[B30] Perisic L. Aldi S. Sun Y. Folkersen L. Razuvaev A. Roy J. (2016). Gene expression signatures, pathways and networks in carotid atherosclerosis. J. Intern Med. 279 (3), 293–308. 10.1111/joim.12448 26620734

[B31] Pierce B. L. Ahsan H. Vanderweele T. J. (2011). Power and instrument strength requirements for Mendelian randomization studies using multiple genetic variants. Int. J. Epidemiol. 40 (3), 740–752. 10.1093/ije/dyq151 20813862 PMC3147064

[B32] Polonsky T. S. McDermott M. M. (2021). Lower extremity peripheral artery disease without chronic limb-threatening ischemia: a review. JAMA. 325 (21), 2188–2198. 10.1001/jama.2021.2126 34061140

[B33] Ratnapriya R. Sosina O. A. Starostik M. R. Kwicklis M. Kapphahn R. J. Fritsche L. G. (2019). Retinal transcriptome and eQTL analyses identify genes associated with age-related macular degeneration. Nat. Genet. 51 (4), 606–610. 10.1038/s41588-019-0351-9 30742112 PMC6441365

[B34] Ritchie M. E. Phipson B. Wu D. Hu Y. Law C. W. Shi W. (2015). Limma powers differential expression analyses for RNA-sequencing and microarray studies. Nucleic Acids Res. 43 (7), e47. 10.1093/nar/gkv007 25605792 PMC4402510

[B35] Roy P. Orecchioni M. Ley K. (2022). How the immune system shapes atherosclerosis: roles of innate and adaptive immunity. Nat. Rev. Immunol. 22 (4), 251–265. 10.1038/s41577-021-00584-1 34389841 PMC10111155

[B36] Shi G.-P. Bot I. Kovanen P. T. (2015). Mast cells in human and experimental cardiometabolic diseases. Nat. Rev. Cardiol. 12 (11), 643–658. 10.1038/nrcardio.2015.117 26259935

[B37] Skrivankova V. W. Richmond R. C. Woolf B. A. R. Davies N. M. Swanson S. A. VanderWeele T. J. (2021). Strengthening the reporting of observational studies in epidemiology using mendelian randomisation (STROBE-MR): explanation and elaboration. BMJ 375, n2233. 10.1136/bmj.n2233 34702754 PMC8546498

[B38] Soleymanjahi S. Blanc V. Molitor E. A. Alvarado D. M. Xie Y. Gazit V. (2023). RBM47 regulates intestinal injury and tumorigenesis by modifying proliferation, oxidative response, and inflammatory pathways. JCI Insight 8 (9), e161118. 10.1172/jci.insight.161118 37014710 PMC10243830

[B39] Taniuchi I. Kitamura D. Maekawa Y. Fukuda T. Kishi H. Watanabe T. (1995). Antigen-receptor induced clonal expansion and deletion of lymphocytes are impaired in mice lacking HS1 protein, a substrate of the antigen-receptor-coupled tyrosine kinases. EMBO J. 14 (15), 3664–3678. 10.1002/j.1460-2075.1995.tb00036.x 7641686 PMC394441

[B40] Thanassoulis G. O'Donnell C. J. (2009). Mendelian randomization: nature's randomized trial in the post-genome era. JAMA 301 (22), 2386–2388. 10.1001/jama.2009.812 19509388 PMC3457799

[B41] Urano T. Castellino F. J. Suzuki Y. (2018). Regulation of plasminogen activation on cell surfaces and fibrin. J. Thromb. Haemost. 16 (8), 1487–1497. 10.1111/jth.14157 29779246 PMC6099326

[B42] Wu T. Hu E. Xu S. Chen M. Guo P. Dai Z. (2021). clusterProfiler 4.0: a universal enrichment tool for interpreting omics data. Innov. (Camb) 2 (3), 100141. 10.1016/j.xinn.2021.100141 PMC8454663 34557778

[B43] Zeller T. Münnich I. A. Windisch R. Hilger P. Schewe D. M. Humpe A. (2023). Perspectives of targeting LILRB1 in innate and adaptive immune checkpoint therapy of cancer. Front. Immunol. 14, 1240275. 10.3389/fimmu.2023.1240275 37781391 PMC10533923

[B44] Zhang B. Liu L. Zhou T. Shi X. Wu H. Xiang Z. (2020). A simple and highly efficient method of IFI44L methylation detection for the diagnosis of systemic lupus erythematosus. Clin. Immunol. 221, 108612. 10.1016/j.clim.2020.108612 33069854

[B45] Zhu Z. Zhang F. Hu H. Bakshi A. Robinson M. R. Powell J. E. (2016). Integration of summary data from GWAS and eQTL studies predicts complex trait gene targets. Nat. Genet. 48 (5), 481–487. 10.1038/ng.3538 27019110

